# Enhancing post-thaw quality of ram epididymal sperm by supplementation of rutin in cryopreservation extender

**DOI:** 10.1038/s41598-023-38022-y

**Published:** 2023-07-05

**Authors:** Abouzar Najafi, Hossein Mohammadi, Seyed Davood Sharifi

**Affiliations:** 1 Department of Animal and Poultry Science, College of Aburaihan, University of Tehran, Tehran, Iran; 2 Department of Animal Science, Faculty of Agriculture and Natural Resources, Arak University, Arak, Iran

**Keywords:** Embryology, Reproductive biology

## Abstract

The purpose of this study was to examine the effect of different rutin concentrations on rams epididymal sperm. A local slaughterhouse provided 50 pair of testes from 25 rams. The testes were sent to the lab at room temperature. Spermatozoa were extracted by suspending portions of cauda epididymis in tris solution. Ram sperm was cryopreserved (in liquid nitrogen) in a tris extender containing rutin at 0, 0.5, 0.75, 1, and 1.25 mM. Rutin showed superior sperm total and progressive motility, beat cross frequency, straight line velocity, velocity average pathway and membrane integrity values at 0.75 and 1 mM. The morphology of the sperm and the superoxide dismutase levels did not significantly change with different treatments. Moreover, rutin at 0.75 and 1 mM was also shown to have the highest level of mitochondrial activity. The results showed ATP, total antioxidant capacity, and glutathione peroxidase levels were significantly greater in the rutin 0.75 and 1 mM groups (*P* < 0.05). Rutin at 0.75 and 1 mM levels had the lowest reactive oxygen species concentrations. Rutin at 0.75 and 1 mM substantially increased the proportion of viable sperm (*P* < 0.05). The lowest amount of apoptosis was observed in 0.75 and 1 mM rutin. Rutin at 0.75 and 1 mM yielded the least significant percentage of dead sperm. It may be inferred that adding 0.75 and 1 mM to the sperm extender can enhance the quality of the epididymal sperm in rams after the cryopreservation process.

## Introduction

Sperm cryopreservation has applications in various fields, including assisted reproductive technology (ART), the selective breeding of animals for desirable genetic traits, and the protection of endangered species^1^. In ART procedures, sperm cryopreservation is commonly used to guarantee the availability of sperm for fertilization. However, the fertilizing ability of frozen-thawed sperm is decreased due to the changes in the physiology, structure, and function of the sperm cell. Despite its advantages in reproduction, cryopreservation negatively impacts the lipid content of membranes, acrosome state, sperm motility, and viability^2^. Cryoinjuries may be caused by osmotic stress, cold shock, intracellular ice crystal formation, chemical stress, an accumulation of reactive oxygen species (ROS), a change in antioxidant defense systems, or a combination of these factors^3^. Oxidative stress occurs when the formation of ROS within the body exceeds its capacity to produce antioxidants. Oxidative stress causes a reduction in the number of gametes, sperm motility, and an increase in the percentage of dead cells. To avoid peroxidative damage to sperm membranes, an efficient antioxidant system is required^4^. Optimal cryopreservation of epididymal spermatozoa, for accidental death in rams and other endangered species, can be crucial in preserving biodiversity. This approach can serve as an effective strategy for maintaining genetic diversity and preventing the extinction of endangered species^5^.

The flavone derivative rutin, which is made up of the flavonol quercetin and the disaccharide rutinose, possesses a wide variety of beneficial biological properties^6^. According to the findings of previous studies, rutin can directly remove ROS by giving electrons to free radicals. In addition, it has been shown to protect somatic cells against oxidative stress (OS) by increasing the levels of antioxidant enzymes such as glutathione (GSH), catalase (CAT), and glutathione peroxidase (GPx)^7^.

According to previous studies, rutin can also detoxify the oxidative stress created in the body as a result of numerous medications and substances^8^. Despite the strong antioxidant activity of rutin in several cell types, which has been linked to its beneficial effects on the cardiovascular system, the kidneys, and the nervous system^9^, there have been few studies on the effects of rutin on mammalian sperm, particularly frozen-thawed sperm. Surprisingly, new studies show that rutin can preserve reproductive function in vivo or in vitro conditions^10^. Based on these findings, it seems that including rutin in the cryopreservation extender might be a promising technique for lowering the risk of sperm being damaged by the freezing process^10^.

In addition, there is a lack of data showing how different concentrations of rutin affect the quality of ram epididymal sperm after the freeze-thawing process. Our study represents the first investigation of using rutin as a cryoprotectant for preserving ram sperm, which emphasizes the novelty and potential impact of our findings in the field of reproductive biology. These results could have practical implications in the development of more efficient cryopreservation techniques for preserving the genetic material of endangered species. As a result, the study aimed to assess the influence of rutin on the post-thawing quality of ram epididymal sperm.

## Material and methods

All animal care protocols were accomplished in accordance with ARRIVE guidelines and the University of Tehran guidelines for Animal Experiments. The Animal Research Committee of the University of Tehran authorized the animal study. A local slaughterhouse (Behin slaughterhouse, Tehran, Iran) supplied 50 pair of testes from 25 rams (4–5 years old). They were kept at ambient temperature while being transported to the laboratory. The method of isolating spermatozoa from the cauda segments of the epididymis was reported by Merati, et al.^11^. Spermatozoa were obtained by suspending portions of cauda epididymis in tris solution. The samples were centrifuged at 700*g* for 10 min; then the sperm suspension was placed in tris solution for 15 min and prepared for the tests. Pooled sperm with a motility of 80% or higher and a total morphological abnormality rate of 10% or less were diluted to a final concentration of 250 × 10^6^ spermatozoa/mL. The basic extender comprised 2.71 g Tris, 1. 4 g citric acid, and 1 g fructose in 100 ml distilled water, containing 7% (v/v) glycerol and 20% (v/v) egg yolk^12^.

Semen with motility > 80% and total abnormalities < 10% were pooled. Semen was extended with each extender being cooled to 4 °C at 2 h and subsequently loaded into 0.25 ml French straws (IMV, L’Agile France). Polyvinyl alcohol powder was used to seal the straws, which were then submerged in liquid nitrogen after being held 4 cm above the temperature of the liquid for 7 min. The straws were thawed at 37 °C for 30 s in a water bath after being frozen for at least 4 weeks, and the sperm were then extracted and analyzed^13^.

### Sperm motility and motion parameters

Sperm Analysis was performed using computer-assisted Sperm Analysis (CASA; Video test, Sperm 3.2; St. Petersburg, Russia). Five random fields were selected for evaluating the motility parameters. The program assessed some factors, including total motile spermatozoa percentage (TM), velocity curvilinear (VCL), percentage of spermatozoa with progressive motility (PM), amplitude of lateral head displacement (ALH), straightness (STR), Straight line Velocity (VSL), average path velocity (VAP), beat cross frequency (BCF), and linearity (LIN) (VSL/ VCL) × 100. The number of spermatozoa evaluated was at least 300^14^.

### Plasma membrane functionality

The hypo-osmotic swelling (HOS) test was used to evaluate plasma membrane functionality in sperm. In brief, 10 µL of semen was mixed with 100 µL of a 100 mOsm/kg hypoosmotic medium (Osmolality was measured by an osmometer) (9 g/L fructose and 4.9 g/L sodium citrate) and incubated for 30 min at 37 °C. After that, a phase-contrast microscope was used to analyze a drop of the incubated sample that had been placed on a slide (Labomed, Lx 400, USA; magnification of at 400). At least 200 spermatozoa from each sample were examined, and the proportion of sperm with a coiled tail was considered as sperm with a functional plasma membrane^15^.

### Sperm morphology

Formalin-fixed sperm samples were examined to determine sperm morphology. Two hundred spermatozoa were analyzed to detect abnormalities. At a magnification 400, the evaluation was carried out using phase contrast microscopy^16^.

### Mitochondria membrane potential (MMP)

Rhodamine-123 (R123) and PI were used to estimate the MMP percentage of sperm, as stated by Mehdipour, et al.^17^. Samples of semen diluted with tris buffer (300 µL) and then the R123 (10 μl, Invitrogen TM, Eugene, OR, USA) solution (0.01 mg/mL) was added and incubated at room temperature for 20 min. Then, sperm suspensions were treated with 10 µL of PI (1 mg/mL). A total of 1 × 10^4^ events were recorded for each sample. Sperm that showed a negative signal for PI and a positive signal for R123 were determined to have mitochondria with high membrane potential.

### Phosphatidyl serine externalization

Phosphatidyl serine externalization is defined as an early sign of apoptotic-like alterations in the sperm sample^18^. The concentration of the sperm samples was adjusted to 1 × 10^6^ sperm/ml after being washed in calcium buffer, and 10 µl Annexin V-FITC (0.01 mg/ml) was added to 100 µl of the sperm suspension. Subsequently, the sperm suspension was treated with 10 µl of propidium iodide (PI; 1 mg/ml) and incubated for at least 10 min in room temperature. Then, the suspension was analyzed by flow cytometry.

### TAC, GPx and SOD assessment

The antioxidant system was analyzed by measuring GPx, Total antioxidant capacity (TAC), and superoxide dismutase (SOD) levels. The TEAC method is a test used to measure the total antioxidant capacity, which is based on the ability of antioxidants to scavenge the ABTS cation radical. For the test, a mixture of 20 µL of the sample, 1 mL of ABTS reagent, and 200 µL of H_2_O_2_ was prepared, and the resulting blue-green color was measured using spectrophotometry at 600 nm. The glutathione peroxidase activity was evaluated by mixing 10 μL of the sample with 500 µL of GPx reagent, 10 µL of buffer, and 4 µl cumene hydroperoxide. The absorption was then measured at a wavelength of 340 nm. The superoxide dismutase activity was evaluated by mixing 50 μL of the samples with 1.7 mL of mixed substrate (Xanthine and Int) and then adding 500 μL of xanthine oxidase. The resulting solution was measured at a wavelength of 505 nm. To undertake spectrophotometric assays of these parameters, Randox™ kits (RANDOX Laboratories Ltd.) and an Olympus AU 400 automatic biochemistry analyzer (Olympus, Tokyo, Japan) were used.

### Determination of ATP in sperm

The approach developed by Mehdipour, et al.^13^ was used to measure ATP. Initially, 5 µL of each sample was diluted in 750 µL of buffer before being pipetted into 190 µL of perchloric acid, and the samples were then centrifuged at 12,600×*g* for 2 min. After transferring the upper phase to a new tube, 10.7 µL of 2 M KCl, 58.7 µL of 1 M KOH, 10.7 μl of saturated tris, and 1 µg/mL of red phenol were added. The last step was to add 100 µL of the reconstituted luciferin-luciferase reagent. The ATP standard was serially diluted to produce standards ranging from 10^–7^ to 10^–12^ M. The amount of ATP was expressed as pmol ATP per 10^6^ sperm.

### Determination of ROS

Evaluation of ROS was carried out by the method of Mehdipour et al.^13^. The samples were incubated for 20 min at 37 °C in 250 µL of PBS before being centrifuged at 300×*g* for 7 min to remove the supernatant. After adding 3 mL PBS, the mixture was centrifuged again at 300×*g* for 7 min. Then, 400 µL of the sample was mixed with 10 µL of luminol, and the tubes were inserted into an Orion II microplate luminometer. The findings were represented as 10^3^ counted photons per minute (cpm) per 10^6^ spermatozoa.

### Statistical analysis

For analyzing the data, the software SAS 9.1 was used. Each ram's sperm variables were tested for normal distribution using the Shapiro–Wilk test. Before analyzing the data, the distribution was normalized using arcsine transformation, and the differences among groups were examined using analysis of variance (ANOVA). For comparing treatment means, the Tukey test was utilized. The data were presented as mean ± SE.

## Result

**Figure 1:**
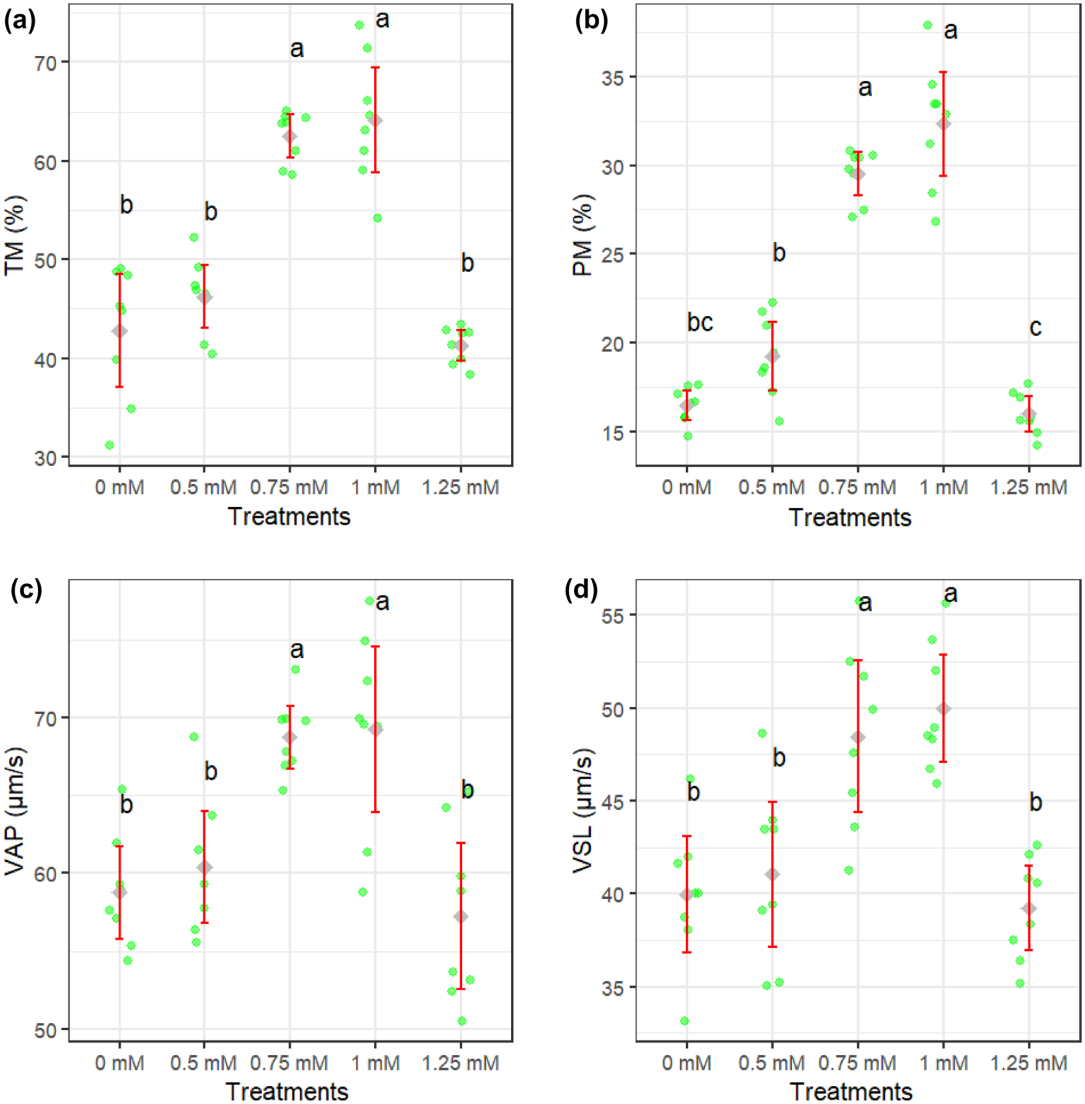
TM: total motility (**a**), PM: progressive motility (**b**), VAP: average path velocity (**c**) and VSL: straight-line velocity (**d**) after thawing samples cryopreserved with different rutin concentrations. Different letters indicate that treatments differ *P* < 0.05.

**Figure 2:**
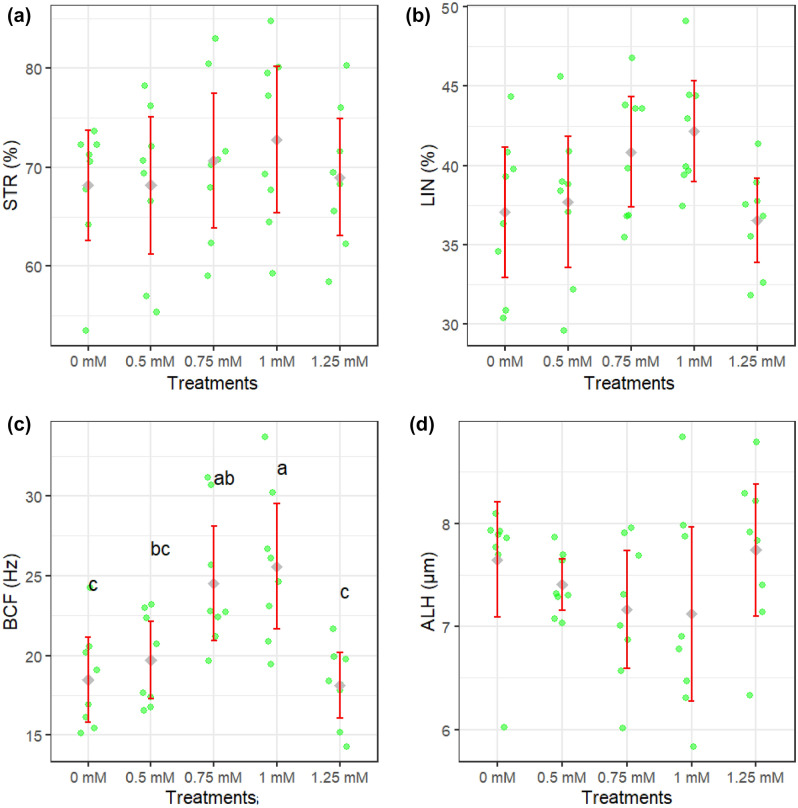
STR: straightness (**a**), LIN: linearity (**b**), ALH: amplitude of the lateral head displacement (**c**) and BCF: beat cross frequency (**d**) after thawing samples cryopreserved with different rutin concentrations. Different letters indicate that treatments differ *P* < 0.05.

**Figure 3:**
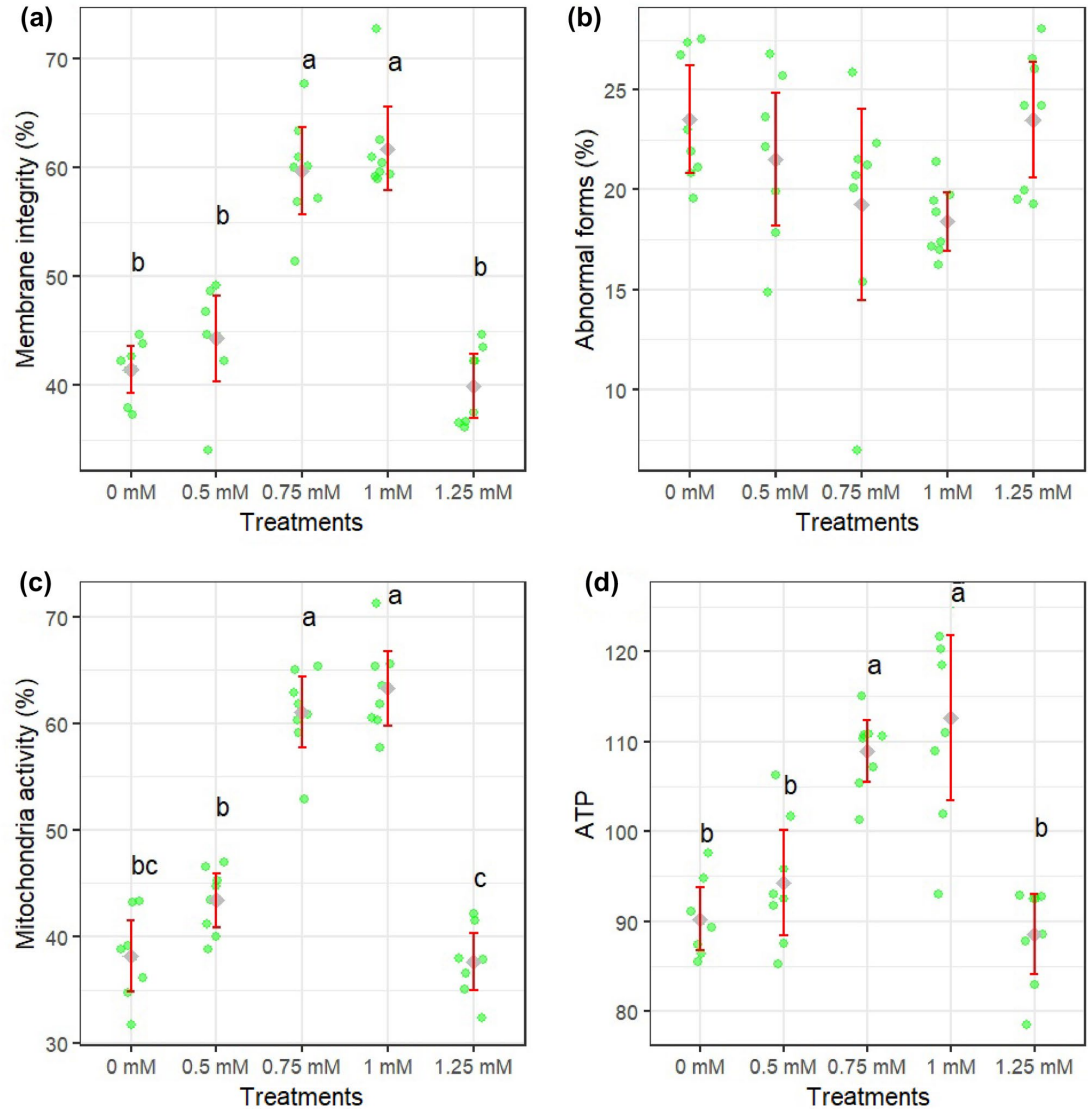
Membrane integrity (**a**), total abnormality (**b**), mitochondrial activity (**c**) and ATP (**d**) after thawing samples cryopreserved with different rutin concentrations. Different letters indicate that treatments differ *P* < 0.05.

**Figure 4:**
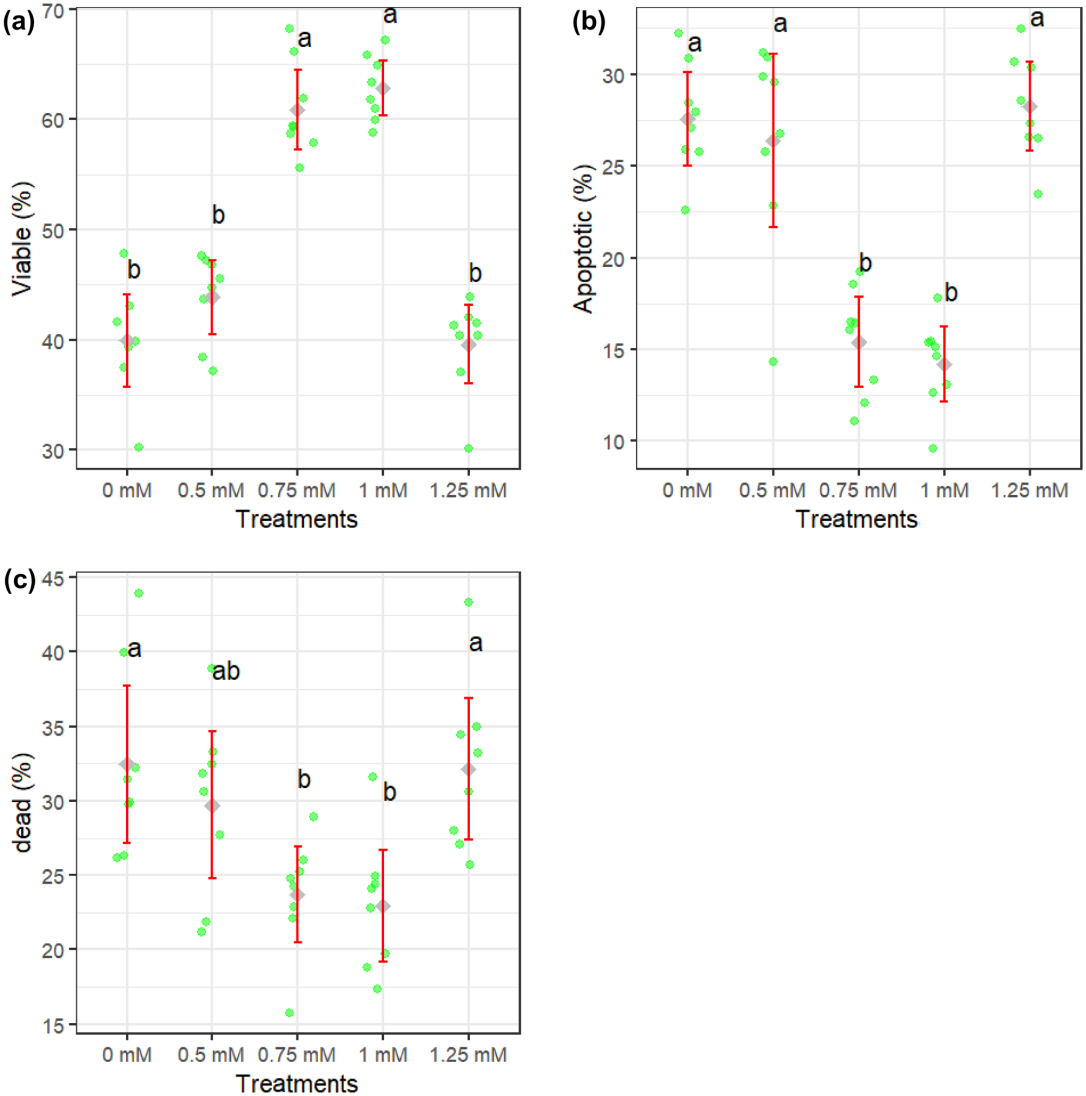
Viability (**a**), apoptotic (**b**) and dead sperm (**c**) after thawing samples cryopreserved with different rutin concentrations. Different letters indicate that treatments differ *P* < 0.05.

**Figure 5:**
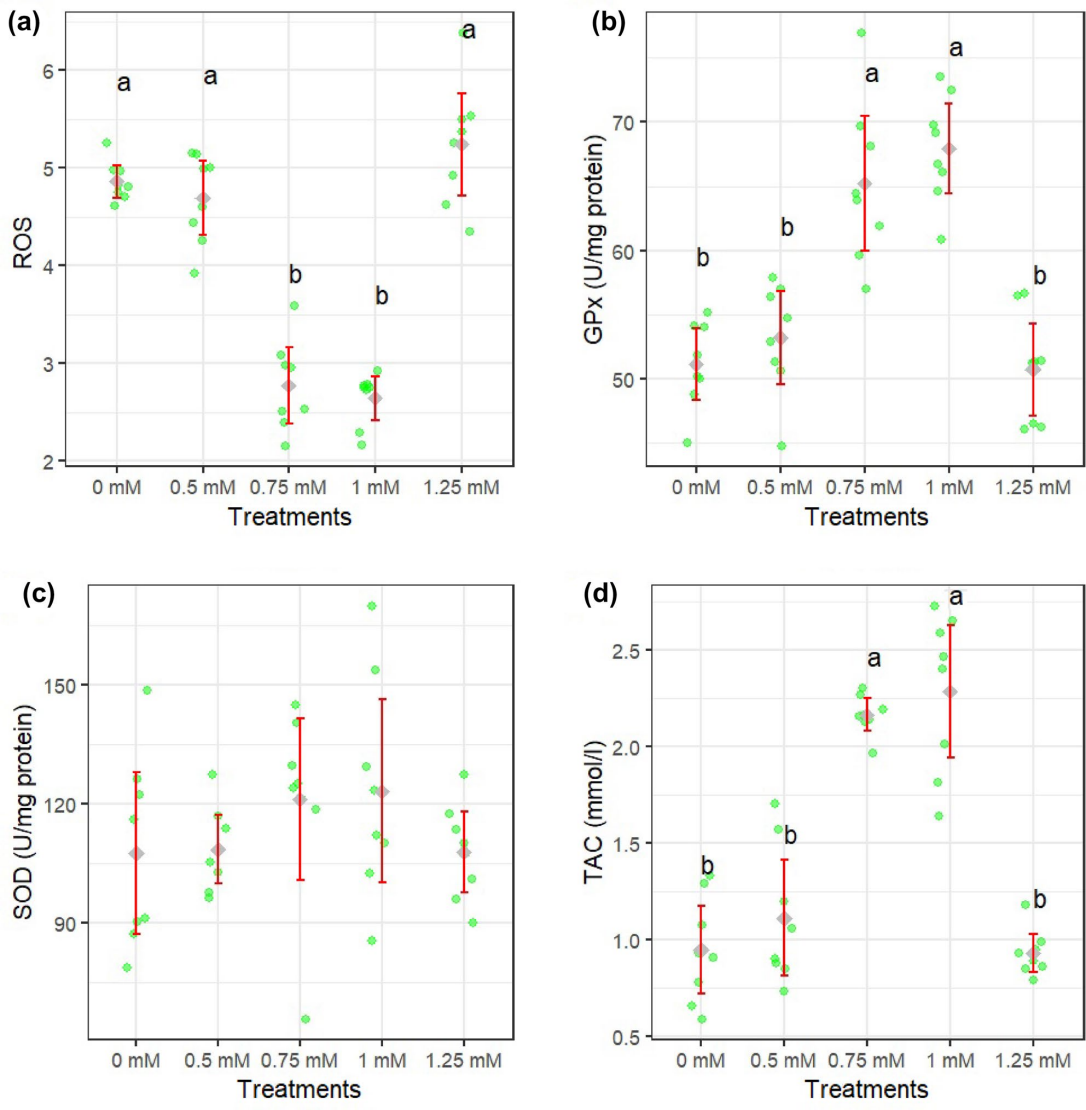
ROS (**a**), GPx (**b**), SOD (**c**), and TAC (**d**) after thawing samples cryopreserved with different rutin concentrations. Different letters indicate that treatments differ *P* < 0.05.

Figures 1a–d and 2a–d show how different concentrations of rutin affect sperm motility after thawing. Sperm total and progressive motility, BCF, VSL, and VAP parameters were improved when extender supplemented by rutin at 0.75 and 1 mM (*P* < 0.05). The adverse effects of rutin on sperm kinematic parameters were attenuated when a supplementation concentration of 1.25 mM was used. Figure 3a–c displays the results of the analysis of the membrane integrity, abnormal morphology, and mitochondrial activity of sperm. Membrane integrity was considerably greater in the rutin group at 0.75 and 1 mM than in other groups (*P* < 0.05). The morphology (Fig. 3b) of the sperm and the SOD levels (Fig. 5c) were the same regardless of treatment. Furthermore, rutin at 0.75 and 1 mM levels exhibited the greatest percentage of mitochondrial activity compared to the other groups (*P* < 0.05). Figure 4a–c depicts the effects of various rutin concentrations on the viability and apoptosis-like alterations in ram sperm following cryopreservation. Groups exposed to 0.75 and 1 mM rutin had a significantly (*P* < 0.05) greater percentage of viable sperm than the other groups (Fig. 4a). At concentrations of 0.75 and 1 mM, rutin inhibited apoptosis to a significantly lower extent than the control. The groups exposed to 0.75 and 1 mM rutin had the lowest percentage of dead sperm.

The rutin at 0.75 and 1 mM levels showed considerably more ATP (Fig. 3a), TAC (Fig. 5d), and GPx (Fig. 5b) than the control group (*P* < 0.05). Compared to the control, 0.75 and 1 mM of rutin produced the least ROS (Fig. 5a).

## Discussion

The sperm quality is believed to be negatively affected by cryopreservation due to two reasons: firstly, the disruption of the plasma membrane, and secondly, malfunctioning of the mitochondria. In the current study, rutin was utilized to prevent the deleterious effects of the freezing and thawing process on ram sperm. The current findings indicated that rutin at 0.75 and 1 mM treatment might effectively alter total and progressive motility, membrane integrity, and viability.

Motility is the primary and the most crucial indicator of sperm quality in all sperm examination techniques. Total and progressive motility, VAP, and VSL of post-thawed sperm were considerably improved in the 0.75 and 1 mM groups in the current study. Our findings are consistent with those of Aksu et al.^19^, who found that treatment with rutin improved rat sperm motility and reduced overall sperm abnormalities. Abarikwu et al.^20^ reported that throughout a 5-week trial with rats, the presence of cadmium led to an increase in the number of defective sperm, as well as a reduction in the motility of sperm and increased sperm count, while rutin eliminated these harmful effects. In another research, compared to the control group, both doses of rutin (5 mg and 10 mg) substantially raised the number of rat viable and motile sperm^21^. Notably, in this study, sperm treated with 0.75 and 1 mM rutin showed a decrease in ROS production while improving viability and membrane integrity. The observed improvements in sperm membrane integrity with rutin (0.75 and 1 mM groups) supplementation may be due to its antioxidant properties, which could help to protect spermatozoa from oxidative damage during cryopreservation. Mata-Campuzano et al.^22^ found that rutin improved membrane integrity in deer spermatozoa following incubation at 37 °C, which is consistent with our findings. Our results suggest that rutin (0.75 and 1 mM groups) may stabilize sperm cell membrane, which is essential for maintaining sperm cell morphology and function. Cryopreservation can cause damage to sperm cell membranes, leading to the loss of membrane integrity and increased susceptibility to damage. Rutin, a polyphenol, may interact with the lipid bilayer of the membrane and provide structural support, thereby increasing the resistance of sperm cells to damage during cryopreservation.

To explore the mechanism of rutin's beneficial effect on post-thaw sperm, we measured the quantity of ROS produced in the frozen-thawed spermatozoa. In accordance with our observations, Xu et al.^23^, found that rutin supplementation significantly reduced ROS accumulation and MDA production while increasing the activities of SOD, CAT, and GSH-Px in post-thaw boar sperm. Khan^24^ observed that adding rutin in using a rat model reduced the adverse effects of ROS and lipid peroxidation on sperm by increasing the activities of antioxidant enzymes. Annapurna et al.^7^ found similar results, reporting rutin increases antioxidant capacity in rat brain cells by stimulating the production of more SOD, CAT, and GSH-Px. Several mechanisms have been identified for how rutin exerts its antioxidant effects. The capacity of rutin to donate electrons, which are a direct target of hydroxyl and superoxide radicals, offers another possible explanation for the antioxidant process facilitated by this compound^25^. These proposed roles for rutin were supported by the current investigation. The higher levels of ATP, TAC, and GPx observed in the rutin at 75 and 1 mM groups may be indicative of improved energy metabolism and antioxidant defense mechanisms in spermatozoa. Apparently, rutin served as a barrier against LPO damage to the sperm membrane. As a result of rutin antioxidant properties, ROS generation and sperm damage were reduced, and sperm quality was enhanced. However, when the higher MDA concentration and lower TAC associated with the control extender group are taken into account, it becomes clear that this latter group experienced a higher LPO rate. This resulted in decreased sperm motility, viability, and membrane integrity, as well as an increase in the proportion of abnormal sperm in the control group. In line with the current findings, it has been proven that low sperm quality, such as impaired sperm motility, is connected with higher levels of MDA^26^.

In the current research, rutin was utilized at levels that appear to be proper for its antioxidant benefits. These findings imply that high concentrations of rutin may impede not just sperm metabolism but also signaling pathways that regulate flagellar beating^22^. Our results suggest that rutin effects on sperm quality may be optimal at specific concentrations. The improvements in sperm quality parameters were observed at concentrations of 0.75 and 1 mM, but not at higher concentrations. This may be due to a saturation effect, where higher concentrations of rutin may not provide additional benefits. Alternatively, higher concentrations of rutin may have detrimental effects on sperm cells, such as toxicity or altered membrane permeability, that offset the potential benefits of rutin supplementation.

In the current investigation, rutin supplementation of ram sperm not only increased mitochondrial activity, but also inhibited apoptosis. The mitochondria are not only the cell energy source but also an essential part of the process that controls apoptosis^27^. Our result suggested that rutin may improve sperm motility and viability by enhancing mitochondrial function. The increased mitochondrial activity in sperm cells treated with rutin suggests that rutin may improve energy production in sperm cells and enhance their motility and viability.

A study found that pre-treatment with rutin protected mice from idarubicin-induced testicular damage^28^. Oxidative stress and high ROS generation cause apoptosis and spermatozoa cell death^29^. Rutin antioxidant activity shows that it is a dietary component that can help prevent male infertility by preserving sperm DNA^30^. Demonstrated the antioxidant properties of rutin to reduce cisplatin-induced sperm damage, testicular degeneration, and apoptosis in adult male rats. Our results indicated that rutin treatment reduced the apoptotic activity produced by high levels of reactive oxygen species. This protective effect might be attributable to rutin ROS scavenging ability. The lower levels of ROS and apoptosis observed in the rutin-treated groups suggest that rutin may have a protective effect on spermatozoa by reducing oxidative stress and preventing cell death.

## Conclusion

In conclusion, the supplementation of rutin at concentrations of 0.75 and 1 mM significantly improves the quality and health of ram sperm following cryopreservation. Rutin supplementation enhances sperm motility, mitochondrial activity, membrane integrity and reduces the production of reactive oxygen species (ROS) and apoptosis. Additionally, rutin supplementation increases the levels of adenosine triphosphate (ATP), total antioxidant capacity (TAC), and glutathione peroxidase (GPx) in sperm cells. These findings suggest that rutin has promising potential as a protective agent for the cryopreservation of ram epididymal sperm.

## Supplementary Information

Supplementary Information.

## Data Availability

The authors declare that the data supporting the findings of this study are available within the paper.
